# Genetic Spectrum of Familial Hypercholesterolaemia in the Malaysian Community: Identification of Pathogenic Gene Variants Using Targeted Next-Generation Sequencing

**DOI:** 10.3390/ijms232314971

**Published:** 2022-11-29

**Authors:** Aimi Zafira Razman, Yung-An Chua, Noor Alicezah Mohd Kasim, Alyaa Al-Khateeb, Siti Hamimah Sheikh Abdul Kadir, Siti Azma Jusoh, Hapizah Nawawi

**Affiliations:** 1 Institute of Pathology, Laboratory and Forensic Medicine (I-PPerForM), Universiti Teknologi MARA, Sungai Buloh 47000, Selangor, Malaysia; 2 Department of Biochemistry and Molecular Medicine, Universiti Teknologi MARA, Sungai Buloh 47000, Selangor, Malaysia; 3 Department of Pathology, Universiti Teknologi MARA, Sungai Buloh 47000, Selangor, Malaysia; 4 Faculty of Pharmacy, Universiti Teknologi MARA, Puncak Alam Campus, Bandar Puncak Alam 42300, Selangor, Malaysia

**Keywords:** familial hypercholesterolaemia, FH, genetic testing, next generation sequencing, pathogenic variants, LDLR gene, APOB gene, PCSK9 gene

## Abstract

Familial hypercholesterolaemia (FH) is caused by mutations in lipid metabolism genes, predominantly in low-density lipoprotein receptor (LDLR), apolipoprotein B (APOB), proprotein convertase subtilisin/kexin-type 9 (PCSK9) and LDL receptor adaptor protein 1 (LDLRAP1). The prevalence of genetically confirmed FH and the detection rate of pathogenic variants (PV) amongst clinically diagnosed patients is not well established. Targeted next-generation sequencing of LDLR, APOB, PCSK9 and LDLRAP1 was performed on 372 clinically diagnosed Malaysian FH subjects. Out of 361 variants identified, 40 of them were PV (18 = LDLR, 15 = APOB, 5 = PCSK9 and 2 = LDLRAP1). The majority of the PV were LDLR and APOB, where the frequency of both PV were almost similar. About 39% of clinically diagnosed FH have PV in PCSK9 alone and two novel variants of PCSK9 were identified in this study, which have not been described in Malaysia and globally. The prevalence of genetically confirmed potential FH in the community was 1:427, with a detection rate of PV at 0.2% (12/5130). About one-fourth of clinically diagnosed FH in the Malaysian community can be genetically confirmed. The detection rate of genetic confirmation is similar between potential and possible FH groups, suggesting a need for genetic confirmation in index cases from both groups. Clinical and genetic confirmation of FH index cases in the community may enhance the early detection of affected family members through family cascade screening.

## 1. Introduction

Familial hypercholesterolaemia (FH) is the most common genetic disorder caused by monogenic mutation in genes related to low-density lipoprotein cholesterol (LDL-C) metabolism, resulting in the severe elevation of serum LDL-C levels, hence increasing the risk for premature cardiovascular disease (CVD). Several FH genes related to LDL-C metabolism have been identified to cause FH, namely, low-density lipoprotein receptor (LDLR), apolipoprotein B (APOB), proprotein convertase subtilisin/kexin-type 9 (PCSK9) and LDL receptor adaptor protein 1 (LDLRAP1) genes. Most FH patients have mutations in the LDLR (90–95%), followed by APOB (5–10%) and PCSK9 (<3%) [1,2]. To date, over 2000 LDLR variants have been reported in the Human Gene Mutation Database [3], implying that pathogenic variants (PV) in LDLR are the most common variants that caused FH.

Generally, individuals with FH may carry PV in one (heterozygous FH, HeFH) or both alleles (homozygous FH, HoFH) [4]. Although both categories experience a lifetime burden of elevated LDL-C and a higher risk of CVD compared to the general population, HoFH subjects are more severely affected. In addition, LDL-C levels can be affected by different genetic defects [5,6] and may also vary based on the presence or absence of polygenic gene variants [7,8]. Pathogenic variants in LDLR may affect the uptake of LDL-C in the blood and therefore can cause the severe elevation of LDL-C. Apolipoprotein B-100 is a ligand attached to the LDL-C particles that are responsible for the LDL receptor–LDL-C binding during the LDL-C uptake. The defects in APOB may affect the binding ability of LDL-C to the LDL receptor, which eventually may also cause FH [4,6]. However, FH subjects affected with the PV of APOB generally have a less severe phenotype compared to FH subjects with PV in LDLR [9]. For PCSK9, it is responsible for LDL receptor degradation in liver cells. Unlike the PV of LDLR and APOB, the pathogenicity of PCSK9 is caused by gain-of-function (GOF) mutation, which results in increasing PCSK9 activity, thus increasing LDL receptor degradation [6]. On the other hand, LDLRP1 encodes a protein that serve as an adaptor for LDL receptor endocytosis in the liver cells, and defects in this gene cause the autosomal recessive form of FH [10]. Although the inheritance mode of the pathogenicity has been described as recessive, Tada et al. had reported that individuals with heterozygous PV in LDLRAP1 usually have mild hypercholesterolaemia [10]. The early detection and treatment of FH patients may reduce the risk of early CVD-related death. Identification of FH subjects is often conducted clinically by utilising the FH diagnostic criteria such as Dutch Lipid Clinic Network (DLCN) and Simon Broome (SB) criteria [11]. To increase the diagnostic accuracy and prompt effective family cascade screening, the European Society of Cardiology and European Atherosclerosis Society (ESC/EAS) guidelines have recommended genetic testing to be conducted in index cases whenever possible [12]. However, genetic testing is not a routine diagnostic protocol in most countries due to the cost factor [13,14].

When LDL-C level alone is not specific enough to be used for FH identification, the genetic testing outcome is one of the most important criteria for a definitive diagnosis of FH, along with a physical examination for tendon xanthomata, corneal arcus and family history [15]. The prevalence of heterozygous FH varies from 1:200 to 1:500 individuals, depending on the population, whilst homozygous FH affects 1 in 360,000 individuals [3,16,17]. Although FH is not rare in the Asian population based on the clinical criteria, >95% of FH patients remain underdiagnosed by the definition of DLCN and SB [13,17]. Furthermore, about 50% of heterozygous FH patients lack obvious phenotypes [18]. Thus, many patients are underdiagnosed and undertreated until suffering from an acute cardiovascular event.

The use of genetic testing for FH diagnosis varies globally [19]. Genetic testing for FH has been performed in many countries such as The Netherlands, Norway, Denmark, United Kingdom, Belgium, Australia, Japan and New Zealand [15,19]. Our group has previously described a 1:100 community prevalence of clinically diagnosed potential FH (definite and probable FH according to DLCN criteria) in Malaysia. Based on the current Malaysian population of 33 million, it is estimated that there are 330,000 FH subjects in Malaysia [20], most of whom are underdiagnosed and undertreated [19]. However, data on the prevalence of genetically confirmed FH in the Malaysian community is still scarce. In addition, some previous reports on variants of FH genes lack data on PV according to international criteria [21,22]. Hence, the spectrum of PV in FH genes in the Malaysian community using NGS remains to be studied.

Several previous studies have focused on the FH patients identified from hospital-based screening or tertiary referral centres, with a lack of reports on community-based clinically diagnosed FH subjects. Furthermore, limited genetic testing methods such as restriction fragment length polymorphism (RFLP), denaturing gradient gel electrophoresis (DGGE), microarray and Sanger sequencing on specific genes were used [12,13,15,16,17,18]. The relatively high 1:100 prevalence of clinically diagnosed FH in Malaysia compared to the estimated worldwide prevalence at about 1:200–300 [23] suggests an urgent need for molecular confirmation amongst these clinically diagnosed patients. Furthermore, several reports demonstrated that NGS is a very reliable screening method for FH mutations in a large number of samples [20,21,22,24]. Therefore, identifying the genetic spectrum of FH PV in the community using NGS will not only verify the high prevalence of clinically diagnosed FH in Malaysia but also helps in understanding the pattern of mutations in Malaysia, which may be different compared to other parts of the world.

In addition, by the genetic confirmation of index subjects, cascade screening can be conducted subsequently, which allows accurate diagnosis and early intervention for affected family members. Therefore, the objectives of this present study were to determine the prevalence genetically confirmed FH and to describe the genetic spectrum and frequency of PV in FH genes amongst clinically diagnosed FH subjects in the Malaysian community.

## 2. Results

### 2.1. Clinically Diagnosed FH Subjects

Table 1 illustrates the clinical characteristics of the clinically diagnosed FH subjects distributed into potential FH and possible FH according to the DLCN criteria. A total of 372 amongst 5130 unrelated community individuals were clinically diagnosed as FH. A total of 58/5130 individuals were categorised as potential FH, which translated to a prevalence of clinically diagnosed FH at 1:100. All FH individuals (*n* = 372), including possible FH, were referred for targeted NGS.

The potential FH were older than possible FH subjects (mean ± SD = 58.9 ± 9.9 vs. 49.6 ± 13.1 years, *p* = 0.01). The presence of existing personal CVD and a family history of PCAD were in higher proportions in the potential compared to the possible FH groups (13.8 vs. 5.1%, *p* < 0.01; 34.5 vs. 13.4%, *p* < 0.001, respectively). The TC and LDL-C levels were higher in the potential FH than the possible groups (7.4 ± 1.6 vs. 7.3 ± 1.0 mmol/L, *p* < 0.001; 6.1 ± 1.4 vs. 5.6 ± 0.7, *p* < 0.001, respectively). The proportions with the presence of tendon xanthomata and premature corneal arcus were higher amongst the potential than the possible FH groups (10.3 vs. 0.0%, *p* = 0.001; 75.9 vs. 2.2%, *p* < 0.001, respectively).

### 2.2. Detection Rate and Prevalence of Genetically Confirmed FH in the Malaysian Community

Table 2 demonstrates the detection rate of genetically confirmed FH according to DLCN categories. Genetic analysis of the four FH genes showed that 82/372 (22.0%) of clinically diagnosed FH subjects were genetically confirmed. Potential and possible FH subjects have an almost similar in-group detection rate of PV at 12/58 (20.7%) and 70/314 (22.3%), respectively. The overall prevalence of genetically confirmed FH in the Malaysian community as defined by having potential FH was 1:427 (0.2%–12/5130), whilst the prevalence of genetically confirmed FH when including possible FH was higher, at 1:63 (1.6%–82/5130) (Table 3).

### 2.3. Genotype Frequencies of FH Pathogenic Variants

Amongst the 82 FH subjects with at least one PV in the FH genes, 31.7%, 22.0% and 39.0% individuals had heterozygous PV in *LDLR, APOB* and *PCSK9* alone, respectively (Table 4). Double-heterozygous *LDLR* + *APOB* and *APOB*+*PCSK9* were found in 3.7% and 2.4% of them, respectively, whilst 1.2% were double heterozygous for each *LDLR* + *PCSK9*.

### 2.4. Distribution of Identified Variants According to the Genes

Four FH genes consisting of *LDLR, APOB, PCSK9* and *LDLRAP1* were sequenced amongst all clinically diagnosed FH patients (*n* = 372). A total of 361 different variants were identified, but only 40 of them were classified as PV, whilst 71 and 250 were classified as VUS and benign, respectively, according to ACMG criteria. Amongst the PV, 18 (45.0%), 15 (37.5%), 5 (12.5%) and 2 (5.0%), were from *LDLR, APOB, PCSK9* and *LDLRAP1*, respectively (Table 5).

### 2.5. List of Pathogenic Variants Identified amongst Genetically Confirmed FH Subjects

Table 6 shows the details of each PV in *LDLR, APOB, PCSK9* and *LDLRAP1* identified amongst clinically diagnosed FH subjects in this study. Amongst these PV, 18 were from *LDLR*, 15 from *APOB*, five from *PCSK9* and two from *LDLRAP1*. Of the 18 *LDLR* PV, 17 were missense variants and one was the splicing site variant, whilst in *APOB*, one was a frameshift deletion PV, and 14 others were nonsynonymous variants. In *PCSK9*, three PV were identified as GOF mutation according to the previous studies, whilst one PV in *LDLRAP1* was a frameshift mutation. Amongst the 18 PV in *LDLR*, 14 PV identified were previously reported either in the database and/or literature, whilst only three were previously reported in Malaysia. The most common PV in *LDLR* was *LDLR*:c.1774G>T (G592W), which was detected in six individuals (baseline LDL-C 5.7 ± 0.9), followed by *LDLR*:c.1284C>G (N428K) and *LDLR*:c.301G>A (E101K) that were detected in four individuals each. The baseline LDL-C of individuals with *LDLR*:c.1284C>G (N428K) was 5.6 ± 0.5 and for *LDLR*:c.301G>A (E101K) was 6.5 ± 0.5 mmol/L. There were also 13 missense variants and one deletion variant in the hotspot exons of *APOB*, which are exon 26 and 29, respectively. In addition, there were also 31 individuals with *PCSK9*:c.1493A>C (E498A) variant in exon 9.

## 3. Discussion

To the best of our knowledge, this study is the first to report the prevalence and frequency of genetically confirmed FH amongst clinically diagnosed FH subjects in the Malaysian community based on the clinical, biochemical and genetic characteristics of the subjects. The present study also included an assessment of the genetic spectrum of FH in Malaysia, particularly the PV of the FH genes in the community setting. In the present study, 372 community subjects met the clinical FH criteria by DLCN, wherein at least one PV in FH genes were identified in 22.0% of all clinically diagnosed FH subjects. The potential and possible FH groups also exhibited a similar genetic detection rate of 20.7% and 22.3%, respectively. If only clinically diagnosed potential FH is taken into account, this study suggests the prevalence of genetically confirmed potential FH in Malaysia as 1:427. However, if all clinically diagnosed FH (including possible FH), the overall prevalence of genetically confirmed FH is much higher at 1:63.

A total of 361 different variants in FH genes were identified amongst the clinically diagnosed FH subjects, but only 40 (11.1%) of these variants were classified as PV, whilst 71 (19.7%) were VUS according to the ACMG criteria. Amongst the PV, 45.0% (18/40), 37.5% (15/40), 12.5% (5/40) and 5.0% (2/40), were from *LDLR*, *APOB, PCSK9* and *LDLRAP1,* respectively. About 32% and 22% of clinically diagnosed FH subjects in this study have a PV in *LDLR* and *APOB*, respectively. Interestingly, in this present study, almost 40% of genetically confirmed FH subjects have PV in *PCSK9* alone, of whom 31 clinically diagnosed FH subjects were identified to have c.1493A>C (E498A) PV located in exon 9. A similar distribution of PV in *LDLR* (45%) and *APOB* (37.5%) in the present study is unique compared to the previously reported variant frequency in other population where the most frequently identified PV of FH genes is *LDLR,* which accounts for 60 to 80% of the PV identified in the population [57].

The detection rate of PV in FH varies depending on the population, screening method and diagnostic criteria tools [1]. In this study, the PV of four FH genes were found in 22.0% of the 372 unrelated clinically diagnosed subjects with DLCN criteria, which was lower than previous studies conducted in other countries, such as that reported in Korea (33%) [48], Japan (57.6%) [58], Taiwan (58.8%) [59], India (47%) [60] and United Kingdom (61.9%) [61]. However, the detection rate of FH PV in this study is higher than in the Latvian population, 7.6% [62]. A study by Vandrovcova et al. that adopted NGS to detect variants in the population reported a higher mutation-detection rate of as much as 67% amongst definite FH subjects [41]. In addition, a study by Maglio et al. showed a detection rate of 65% amongst the participants with a family history of CAD, with the majority classified as potential FH and about 20% had xanthomata [63]. The stark different detection rate of PV amongst clinically diagnosed FH between this present study and Maglio et al. (22.0% vs. 65%) could be explained by the proportions of subjects with possible FH and potential FH by DLCN; Maglio et al. reported 74% rate amongst potential FH subjects involved in their study [63], whilst in this present study, the potential FH subjects undergoing genetic analysis constituted only 15.6% (58/372) of the total. Furthermore, the inconsistency of the results between the present study and other studies in different populations could be due to several reason, for example, different NGS methods being used or different LDL-C cut-off levels as one of the inclusion criteria, as well as the different diagnostic criteria used [64]. In addition, with much stronger inclusion criteria such as clinical signs of FH or a strong family history of CAD, these may contribute to the higher detection rate of FH PV. Furthermore, the prevalence of polygenic hypercholesterolaemia genes in a population may also affect the detection rate of PV in the FH genes. In this present study, a lower LDL-C cut-off was used at 4.0 mmol/L, which probably explains the higher proportion of possible FH for genetic analysis. However, it is interesting to note that detection rates of genetic confirmation were similar between potential and possible FH groups (20.7.0% vs. 22.3%), suggesting a need for genetic confirmation in index cases from both groups in clinically diagnosed FH in the Malaysian community. This is perhaps unique in the Asian region where the *APOB* PV is also common, along with *LDLR,* and presents as milder clinical phenotypes classified by DLCN as possible FH. However, it is important to identify and confirm them in index cases and family members due to lifetime exposure to hypercholesterolaemia; hence, if left undiagnosed and untreated, they are subjected to increased PCAD risk.

Our group has previously reported from the same cohort as the present study a 1:100 prevalence of clinically diagnosed potential FH by DLCN in the Malaysian community [20], which was much higher than the FH prevalence in the global population, wherein it is about 1:200–300 [65]. The reported prevalence of clinically diagnosed FH in the Malaysian community was amongst the highest, along with another FH population-based study in Russia that reported a prevalence of clinically diagnosed FH at 1:108 [66]. A summary on the prevalence discrepancies of genetically confirmed FH in general population amongst different countries is illustrated in Table 7. This present study has established a prevalence of 1:427 of genetically confirmed FH amongst the potential FH in the Malaysian community, which was lower compared to other countries, such as in the UK and the U.S population. However, the prevalence of genetically confirmed FH of this present study is much higher compared to Japan, Denmark, Finland and Switzerland.

Despite the higher prevalence of clinically diagnosed FH in the Malaysian population, a large proportion of those subjects are unconfirmed by the NGS. This could be explained by some factors, including PV, that could be located in the uncovered regions by NGS [72], polygenic cause of hypercholesterolaemia [7], effects of high lipoprotein(a) levels instead of true FH [73], or mixed dyslipidaemia [74]. With the current detection rate of PV in this present study, it is likely that the majority of the clinically diagnosed FH subjects who are without any PV in FH genes may have polygenic hypercholesterolaemia. Furthermore, the proportions of polygenic hypercholesterolaemia is often reported to be high amongst mutation-negative FH patients [7,75].

More interestingly, this present study has also clearly demonstrated that, if all categories of FH (potential and possible FH) were included for genetic analysis in this population, the genetically confirmed prevalence is further increased to 1:63. Furthermore, this present study highlighted that one-fourth of potential FH, and similarly, about one-fourth of possible FH had genetic confirmation of at least one PV in FH genes in this community and possibly in other parts of the Asian population with similar genetic profiles. These suggest a potential need for genetic screening for even possible FH and lower LDL cut-offs, which may have been missed if higher LDL-C cut-offs are practiced.

Low-density lipoprotein cholesterol receptor is responsible for clearance of lipoproteins from the blood, where it binds to the apoB ligand on the LDL-C particles and forms the LDL-C–ligand receptor complex. This LDL–ligand receptor complex transports LDL-C into the cells via receptor-mediated endocytosis, hence reducing the LDL-C in the blood. Genetic defects in *LDLR* will cause a decrease in LDL receptor functions at different levels, such as LDL receptor not being synthesised, the partial or complete retention of receptor in the endoplasmic reticulum, the inability to bind with the apoB ligand, defective endocytosis and diminished receptor recycling capacity [76]. On the other hand, mutation in *APOB* causes inability to bind to receptor, whilst GOF mutations in *PCSK9* will increase PCKS9 activity and cause receptor to be targeted for degradation [77]. For *LDLRAP1*, it encodes an adapter protein that binds to clathrin, which is responsible for facilitating the internalisation of the LDL–LDL receptor complex. The loss of function in the *LDLRAP1* prevents the internalisation of this complex [78]. Therefore, mutations in the any of the above genes may affect LDL-C metabolism. The genetic spectrum of FH differs significantly across nations. In the present study, 40 different PV of FH genes were identified in 82 subjects. Of the 40 PV identified, 45.0% were PV in the *LDLR* gene, followed closely by *APOB* (37.5%), whilst PV in *PCSK9* and *LDLRAP1* were 12.5% and 5.0%, respectively. In addition, a total of 32 FH subjects had PV in the *PCSK9* gene alone, whilst another six of the FH subjects had double-heterozygous PV.

In most populations, there is a wide spectrum of *LDLR* variants, of which the PV in the *LDLR* variant is the most common cause of FH. A total of 18 *LDLR* PV were identified in 30 clinically diagnosed FH subjects in this present study, of which 14 variants were previously reported either in the literature or public database. Of that 14 reported PV, only three *LDLR* variants (*LDLR*:c.241C>T; c.301G>A and c.1187-2A>G) have previously been reported in Malaysia [21,28,45].

In this study, the *LDLR*:c.241C>T (p.Arg81Cys) located in exon 3 (ligand-binding domain) was classified as pathogenic and has been identified in one FH subject. This variant involved conserved nucleotide changes that eventually replace the large and basic arginine with a medium-size and polar cysteine at codon 81. This variant has also previously been discovered in African [78], Italian [79], Venezuelan [80], Dutch [25] and Portuguese populations [81]. In silico findings from the present study were in agreement with other studies, wherein this variant was predicted to be damaging and to impact protein functioning [25,27]]. This variant was reported as a PV in a Singaporean report based on the in silico prediction [27]. An individual with this variant usually has modest elevation of LDL-C levels [26]. Graca et al. (2022) reported this variant as likely pathogenic, but the functional results were borderline in terms of major impact on the *LDLR* activity [82]. The index FH subject of this study had a baseline LDL-C of 6.2 mmol/L, which is highly suggestive that this variant could affect the *LDLR* activity.

The *LDLR*:c.301G>A (p.Glu101Lys) located in the ligand-binding domain of exon 3 has been found in four individuals in this present study (mean ± SD of baseline LDL-C: 6.5 ± 0.5 mmol/L). Al-Khateeb et al. (2011) reported that the missense mutation of this variant caused a change in glutamate to lysine [28], of which the changes occur in the second disulfide-rich repeat in the receptor protein binding domain, affecting the newly synthesised protein [28]. Apart from this study, this variant was also identified in >30 FH subjects in other previous studies [36,58,61] and co-segregates in two families in the United Kingdom [83,84]. Thormaehlen et al. (2015) conducted a functional study and have discovered that this variant significantly inhibits LDL-C uptake in cells, which eventually impacts protein function [85]. Furthermore, the in silico findings from this present study is highly suggestive of a damaging outcome and is also consistent with other studies [28,86].

A splice site PV, *LDLR*:c.1187-2A>G located at the intron 8/exon 9 junction, has been identified in this present study. This variant destroys the canonical splice acceptor site, which is located in the intron 8/exon 9 junction, which causes abnormal gene splicing and loss of function [87]. The abnormal gene splicing reduces *LDLR* activity to around 40% of normal levels [87,88]. In this present study, this heterozygous variant was identified in a FH subject with very high baseline LDL-C of 9 mmol/L and had corneal arcus. Interestingly, the FH subject with this splice site PV has a higher LDL-C level than those with missense PV.

The remaining 11 *LDLR* variants identified in this study had been described elsewhere in other studies covering Asian, European and American regions. However, these variants were reported for the first time in the Malaysian community. These include two variants located in the ligand-binding domain (1) *LDLR*:c.580A>G [rs373488885, exon 4]; (2) c.811G>A [rs749220643, exon 5], eight variants located in the EGF precursor homology domain: (1) c.949G>A, [rs746834464, exon 7]; (2) c.1234A>C [rs1225797407, exon 9]; (3) c.1284C>G [rs368708058, exon 9]; (4) c.1571T>G [exon 10]; (5) c.1820A>G [rs879255033, exon 10]; (6) c.1867A>G [rs555292896, exon 13]; (7) c.1217G>A [rs552422789, exon 9]; (8) c.1246C>T [rs570942190, exon 9] and one variant in the membrane spanning region: c.2530G>A [exon 17]. Interestingly, four newly discovered variants were reported for the first time by this study and were not reported elsewhere. These includes *LDLR*:c.833G>A [exon 6], c.1289T>G [exon 9], c.1774G>T [exon 12] and c.2383C>G [exon 16]. This highlight an important finding from this study, of which these four PV of *LDLR* could be potential novel variants that currently have been identified only in the Malaysian population.

The number of PV in *APOB* in this present study is almost similar to that in *LDLR*; this suggests that both the *LDLR* and *APOB* genes are common in the Malaysian population. A previous study has shown that variants in the *APOB* gene are a common genetic cause of FH in Taiwan [89]. In Denmark, the FH mutations were mainly in *APOB* and *LDLR* [90]. Other Malaysian studies have shown that *APOB* mutations are also common in FH subjects. Al-Khateeb et al. (2013) reported 10 *APOB* variants were found amongst 30 FH subjects [22], whilst Alex et al. (2012) found 73 *APOB* variants from 137 of total variants amongst 140 genetically confirmed FH subjects [21]. However, those earlier reports did not clearly define the pathogenicity of the discovered variants with any standardised guidelines, whilst this present study determined the FH variant pathogenicity using standard guidelines according to the ACMG criteria [91]. Furthermore, the pathogenicity of FH variants in the Asia Pacific region is not well established. To the best of our knowledge, this is the first FH study in Malaysia that utilises targeted NGS for genotyping and the pathogenicity of variants is being determined using internationally recognised guidelines.

Of all 15 PV of *APOB* that had been identified in this present study, only three of them had previously been reported in other literatures, namely *APOB*: c.13028_13029del (rs760832994)*,* c.10579C>T (rs144467873) and c.8462C>T (rs72653095). This present study identified *APOB*:c.13028_13029del (exon 29) in four possible FH subjects (Baseline LDL-C:5.6 ± 0.9 mmol/L). The deletion of one nucleotide at that particular DNA sequence caused a frameshift alteration at the last 221 amino acids and had also been reported in a Malaysian individual affected with mild hypercholesterolaemia [50]. Although Al-Khateeb et al. reported this variant as VUS, recent evidence in ClinVar has reported this variant as likely pathogenic. Furthermore, in this present study, this variant fulfilled the criteria as PV (PVS1, PS4, PM1, PM2, PP4, PP5) according to the ACMG guidelines.

Another two previously reported PV were described and reported for the first time in Malaysian population through this present study. *APOB*: c.10579C>T was identified in one possible FH subject with a baseline LDL-C of 4.9 mmol/L. This variant was reported to be a common variant in the Asian region [47,92,93] and to co-segregates in multiple families with the same disease [94]. In addition, functional studies had demonstrated that this variant reduced *APOB* capacity for the binding, uptake and degradation of LDL-C [95]. In silico analysis for this variant in this present study is positive, and this is in parallel with another study that reported that this variant may have a deleterious impact on its protein structure and function [96]. *APOB*: c.8462C>T (rs72653095) was reported previously in the European American population [49]. Although it was not reported as pathogenic according to the database, the in silico analysis from this present study suggests the variant has damaging and deleterious effects on the protein function. From the remaining 12 PV of *APOB* identified in this present study, five variants were reported only in the database, without any literature finding, whilst another seven were newly discovered variants described for the first time in this present study.

Proprotein convertase subtilisin/kexin type 9 gene encodes for the PCSK9 protein and comprises a signal peptide (1–30 amino acids), a prodomain (amino acids 31–152), a catalytic doman (amino acids 153–421) and a C-terminal domain (amino acids 422–692) [97]. Genetic variation in *PCSK9* has an enormous impact on the LDL-C levels and has been described either in loss-of-function (LOF) mutation that cause hypocholesterolaemia or GOF mutation, which is associated with FH [98]. The GOF mutations of *PCSK9* usually consists of missense variants located in any exon except exon 3 [98]. In the present study, a total of three GOF PV (Table 6) located in exon 2 of *PCSK9* were identified in this study and were described for the first time in the Malaysian community: *PCSK9*: c.323T>G (rs1057519691), c.212C>T (rs569379713) and c.286C>T (rs185392267). *PCSK9*:c.323T>G has been reported previously in France population [51]. Experimental studies have shown that this missense variant affects *PCSK9* function [51,99]. The variant has been identified in one definite FH subject with a baseline LDL-C of 6 mmol/L and who has a personal history of CAD and the presence of tendon xanthomata. *PCSK9*: c.212C>T variants substitute proline to leucine at codon 71. This variant was suggested to have a GOF effect based on the identification of this variant in two patients who have CAD, stroke and hypercholesterolaemia [52,99]. However, in this present study, the in silico predictions are in conflict, with PolyPhen and REVEL predicting a benign nature, whilst SIFT predicts it to be deleterious. The two FH subjects with this variant have a mean baseline LDL-C of 5.7 ± 0.3 mmol/L without a personal nor family history of CAD and stroke. *PCSK9*: c.286C>T has been identified in one FH subject in this study with a baseline LDL-C of 5.2 mmol/L. Hopkins et al. (2015) also identified the presence of this variant in three FH subjects with a comparable baseline LDL-C (4.9 ± 0.9 mmol/L) [53]. Two out of three in silico tools in this present study predicted that this variant has a damaging or deleterious effect on the protein function. Furthermore, the pathogenicity of this variant was supported with a functional study by Elbitar et al., which has proven that this variant may lead to an increase in LDL receptor degradation [54]. In addition, all three identified *PCSK9* PV with GOF located in exon 2 of *PCSK9,* suggesting that exon 2 is a hotspot exon for the *PCSK9* mutation.

To the best of our knowledge, this study is the first to report the detection and identification of pathogenic *PCSK9* variants (located in exon 9), namely *PCSK9*: c.1493A>C (E498A) and c.1495C>G (R499G), in the community, which have not been described in Malaysia or globally. These variants are located in the C-terminal domain of *PCSK9*, which is responsible for increasing the affinity between PCSK9 protein and the LDL receptor at the low pH of the endosomes [97]. The *PCSK9*: c.1493A>C (E498A) has evidence of likely pathogenicity (ACMG: PM1, PM2, PP3 and PP4) and was found in 31 individuals, whilst a majority of the subjects (27/31) did not have any other PV in other FH genes reported in this study. Furthermore, the in silico findings in this present study highly suggest a damaging effect of the variant that could affect protein function. Several variants in exon 9 of *PCSK9* are projected on the crystallographic structure (Supplementary Materials Figure S1). To support the pathogenicity of *PCSK9*: c.1493A>C (E498A), we have conducted a structural analysis of E498A and projected it on the crystallographic structure of the *PCSK9* (PDB ID 3P5C). Based on this experimental structure, the wildtype residue, E498, is shown to form hydrogen-bond interactions with two serine residues, S488 and S564. However, for the E498 variant, computational mutagenesis predicts the replacement of glutamic acid (E), which is a negatively charged amino acid to an alanine (A), a small hydrophobic amino acid that cause a loss of interaction with one of the serine molecules. Hence, we deduced that the disruption of the intramolecular interactions may affect the overall structural conformation of the *PCSK9* C-terminal domain (Supplementary Materials Figure S2).

*PCSK9*:c.1495C>G (R499G) was found in four subjects, and this variant has pathogenicity evidence of PM1, PM2, PM5 and PP4, which classified this variant as likely pathogenic according to the ACMG guidelines. Initially, the in silico finding of this variant from this present study revealed that it is less likely to be damaging. However, based on the experimental structural analysis of the wild-type, R499 is shown to form salt-bridge (ionic) interactions with E501 and hydrogen bonds with R510. Computational mutagenesis of R499G revealed that the ionic interaction between the variant and residue E501 is abolished due to the change of amino acid. The same scenario occurs with the previously reported PV of *PCSK9*, R499H [98]. The abolishment of the ionic interaction is highly likely to disrupt the structure of the C-terminal domain of *PCSK9* and hence may affect protein function (Supplementary Materials Figure S3). Therefore, for these two variants, molecular docking analysis is warranted in order to explore the impact and the roles of these potential PV of *PCSK9* in FH. 

Additionally, based on the structural analysis, another variant, R496W variant caused loss of interactions with two residues. The replacement of tryptophan, which is hydrophobic and has a bulky aromatic side-chain may cause a collision with nearby residues that highly likely will disrupt the structure of the C-terminal domain of *PCSK9* In contrast, structural analysis were also conducted on a few reported variants (Supplementary Materials Figure S4). On the other hand, structural analysis of neighboring variants in exon 9 of *PCSKS9* such as P467A, R469W, I474 and A478T shows that the wildtype amino acids of these variants have no polar interaction with the surrounding amino acids. Thus, there will not be much impact on the structure caused by the variants, especially for the I474V and A478T variants that are replacing similar types of amino acids (Supplementary Materials Figure S5). 

Two heterozygous variants of *LDLRAP1* were identified in this study, namely *LDLRAP1*:c.281C>A (exon 6) and c.604delT (exon 6). Although no literature was found for the first variant, two out of three in silico tools predicted that *LDLRAP1*:c.281C>A has a damaging effect. As this variant, to the best of our knowledge, has not been reported in any literature or database and hence is likely to be novel in the Malaysian population and worldwide. For *LDRAP1*:c.604delT, the deletion of the nucleotide can cause frameshift mutation. The pathogenicity of this variant is supported by two literature findings that reported this variant as a PV [55,56]. Therefore, *LDRAP1*:c.604delT was classified as PV for this present study, with a potential caveat that the phenotypic presentation is only expected when variants are presented in homozygous forms, given its autosomal recessive mode of inheritance. However, several studies have reported that subjects affected with heterozygous *LDLRAP1* variants were associated with mild hypercholesterolaemia [10,100], but the effect of those heterozygous *LDLRAP1* variants can only be definitively confirmed through extensive family screening and functional studies [10,100]. These *LDLRAP1* variants, however, have not been previously reported in the Malaysian population and thus are likely to be novel variants in the Malaysian population.

Amongst the 372 clinically diagnosed FH subjects, 290 individuals were PV-negative for FH genes. This negative result may be due to the mutations that are rare and yet to be identified in genes other than *LDLR*, *APOB*, *PCSK9* and *LDLRAP1*. In addition, it could also be due to polygenic hypercholesterolaemia, as they may inherit several small-effect polygenic genes that cause the cumulative elevation of LDL-C. Nevertheless, in the absence of PV, these PV-negative subjects are still at a higher risk of atherosclerotic cardiovascular disease [101,102].

Genetic testing for FH has been performed and used in many countries such as Denmark, the Netherlands, the United Kingdom, Spain, Australia, Japan and Korea. Studies involving genetic testing for FH have proven that this method is of great utility for identifying FH subjects with unclear clinical diagnosis, who were already at a higher risk for PCAD. This present study, which sequenced the whole coding regions of the four most popular FH genes rather than only certain known mutations, allowed for the identification of more potential PV not only present in the hotspots but also in other regions of the genes. Furthermore, the identification of different PV in FH genes in this study amongst 372 clinically diagnosed FH subjects in the community may prompt family cascade screening for the index cases with PV, which may offer opportunities for the early detection and treatment of affected family members. In addition, genetic confirmation may assist improvement in FH management in terms of the treatment, wherein the identification of PV in the clinically diagnosed FH subjects may justify the use of more potent and expensive newer lipid-lowering medications for achieving LDL-C targets.

The present study does not perform functional analysis to validate the pathogenicity of the identified variants. Although the main candidate genes for FH have been included, about 78% of the clinically diagnosed FH subjects did not have PV in any one of the four FH genes. Since the recruitment of the FH subjects was from the community health screening programmes, some participants were not able to provide accurate information for family history and lipid stigmata information. Thus, these factors may affect the clinical diagnosis of FH.

The findings suggest that Malaysia has a low detection rate for FH PV in the general population. The majority (about 78%) of the clinically diagnosed subjects in this study have the absence of PV detected within the four FH genes. These are possible causes of polygenic hypercholesterolaemia or could also be explained by PV that are not within the NGS gene panel used in this study. A differential test may also distinguish FH from other lipid disorders, such as sitosterolaemia, as the treatment between FH and sitosterolaemia are different. Future studies are warranted to address the impact of polygenic hypercholesterolaemia genes in these clinically diagnosed FH patients without genetic confirmation. Polygenic risk scoring will be useful in explaining the genetic cause of hypercholesterolaemia amongst FH patients without any PV in monogenic FH genes. Such tests and methods mentioned above are important, as those genetically confirmed FH subjects have a 4.5- to 10-fold increased risk of coronary heart disease (CHD) [103]. Confirmation of the diagnosis may suggest appropriate treatment and management, including the need for family cascade screening. Furthermore, the VUS of the various FH genes with a strong suggestion of PV may need to be carefully examined further with molecular docking simulation, binding affinity and functional studies, particularly when they have not been previously reported. In addition, it is also important to determine the optimal LDL-C threshold and other clinical data that will provide the optimal prediction of genetic confirmation in clinically diagnosed FH patients, such as through artificial intelligence and machine learning processes. In addition, subsequent family cascade screening of index cases with PV should be conducted for early detection and optimal treatment amongst affected family members in a concerted effort in the prevention of premature cardiovascular disease. A national FH registry needs to be established by incorporating data collection that includes all PV identified in clinically diagnosed FH. *LDLR* and *APOB* PV are common amongst clinically diagnosed FH in the Malaysian community, and the potential and possible FH groups, each demonstrating a similar genetic detection rate of about 21–22%, suggest a potential need for genetic confirmation in index cases from both groups of clinically diagnosed FH. Whether this is unique in Malaysia and possibly some other countries of the Asian region remains to be addressed in future studies.

## 4. Materials and Methods

### 4.1. Study Design and Populations

This cross-sectional population-based study involving 5130 participants was part of the Malaysian Health and Wellbeing Assessment for Familial Hypercholesterolaemia (MyHEBAT-FH), a national epidemiological study, conducted in 2011–2017 [104]. All individuals gave written informed consent, and were voluntarily recruited by convenient sampling, from community health screening programmes conducted in 11 states of Malaysia, representing all regions of the country including east and west Malaysia. Malaysian citizens aged ≥18 years old were included in this study, whilst those with secondary hypercholesterolaemia (hypothyroidism, chronic kidney disease, nephrotic syndrome and cholelithiasis) or pregnancy were excluded.

Details on various information, such as demographic data, medical history, smoking habits, personal history of premature coronary artery disease (PCAD) and family history of PCAD, were collected via face-to-face interview using a standardised questionnaire. Blood pressure (BP) was measured with the subjects seated and taken after at least 5 min of rest. The mean of two systolic and diastolic BP readings was reported as the final BP values of the subjects. The waist and hip circumference were measured using measuring tape. Physical examination for the presence of xanthomas, xanthelasma and corneal arcus was conducted by clinicians.

Familial hypercholesterolaemia was clinically diagnosed using DLCN [105]. The minimum baseline pre-treatment LDL-C for the inclusion of subjects with and without PCAD were set at ≥4.0 mmol/L and ≥4.9 mmol/L, respectively. Potential FH was defined as those with a DLCN score of ≥6 for probable FH and >8 for definite FH. Those with a DLCN score of ≥3 to 5 were categorised as possible FH.

### 4.2. Research Ethics

Ethical approval was obtained from the Institutional Research Ethics Committee (Reference number: UiTM 600-RMl [5/1/6)]) prior to the commencement of the study, which was in accordance to the Declaration of Helsinki. Written informed consent was obtained from all participants prior to their recruitment into the study. The study information sheet highlighted the participants’ rights to voluntarily participate and withdraw from the study at any time without any reason or penalty.

### 4.3. Sample Collection

A total of 10 mL venous blood samples were drawn, divided into plain, EDTA and fluoride oxalate tubes. Serum and plasma were separated from whole blood collected in plain and fluoride oxalate tubes within 2 h of collection by centrifugation at 4000 rpm for 10 minutes for biochemical tests. All biochemical analyses for serum total cholesterol (TC), triglycerides (TG), LDL-C, HDL-C and plasma glucose were analysed using an automated biochemical analyser (COBAS Integra® 400, Roche Holding AG; Basel, Switzerland). The LDL-C concentration was calculated using the Friedewald equation [106]. Genomic DNA (gDNA) was extracted from whole blood collected in EDTA tubes using MasterPure™ DNA Purification Kit for Blood Version II (Epicentre, Madison, Winsconsin) according to the manufacturer’s protocol. The DNA samples were stored at −20 °C until further use.

### 4.4. Targeted Next Generation Sequencing

The concentration and purity of extracted DNA were determined using a SpectraMax^®^ QuickDrop™ micro-volume spectrophotometer (Molecular Devices, San Jose, CA, USA) followed by a Qubit™ fluorometer (Thermo Fisher Scientific, Wilmington, DE, USA). Genomic DNA was also subjected to electrophoresis in 1% agarose gel to confirm the presence of the extracted DNA. The qualified samples were then prepared for the targeted next-generation sequencing (TNGS) covering all the exons (including the exon–intron boundaries), introns and the 5′ and 3′ untranslated regions of the four FH genes (*LDLR, APOB, PCSK9* and *LDLRAP1*). Library preparation was performed using an AmpliSeq Library PLUS kit (Illumina, San Diego, CA, USA), and enriched samples were sequenced on an ISeq 100 sequencer platform (Illumina, San Diego, CA, USA) according to the manufacturer’s instructions.

### 4.5. Bioinformatics Analysis

A GRCh37 hg19 human reference assembly was used to map genomic sequencing data using in the proprietary BaseSpace Sequence Hub application (Illumina, San Diego, CA, USA). A Germline Variant Caller (Illumina, San Diego, CA, USA) was used to perform variant calling, and data were generated in the VCF format. All identified variants were filtered, and variants with minor allele frequency (MAF) >5% in the 1000 Genomes (1000 G) database were considered common variants and were excluded from the analysis. The remaining variants were assessed using (1) in silico web-based software, namely Polymorphism Phenotyping V2 (PolyPhen2), Sorting Intolerant From Tolerant (SIFT) and Rare Exome Variant Ensemble Learner (REVEL); (2) ClinVar and Leiden Open Variation Database (LOVD); (3) journal publications; and (4) clinical data from the FH subjects. The pathogenicity of the variants was determined based on the American College of Medical Genetics and Genomics (ACMG) guidelines [91], which classified variants as pathogenic, likely pathogenic, benign, likely benign or variants of uncertain significance (VUS). Likely pathogenic and pathogenic variants were collectively referred to as PV.

### 4.6. Statisctical Analysis

The statistical analysis was performed using SPSS version 26. The outcome of continuous variables with normally distributed and non-normally distributed data were presented as mean±standard deviation (SD) or medians with interquartile range, respectively. Categorical variables were presented as percentages. Continuous data that were normally distributed were analysed using one-way ANOVA, whilst non-normally distributed data were analysed using a Kruskal–Wallis test followed by a Mann–Whitney test. A chi-squared test was used to test the difference between categorical data. A statistical *p*-value of <0.05 was taken as the cut-off to indicate a significant difference.

## 5. Conclusions

The prevalence of genetically confirmed potential FH in the Malaysian community is 1:427, with about a 21.0% genetic detection rate within the potential FH group. Taking into account all categories of FH, the prevalence of genetically confirmed FH is much higher, at 1:63. The potential and possible FH groups each demonstrated a similar genetic detection rate of about 21.0–22.0%, suggesting a potential need for genetic confirmation in index cases from both groups of clinically diagnosed FH in the Malaysian community. Amongst the PV of FH genes in these clinically diagnosed FH patients, the majority were *LDLR* (45.0%), and *APOB* (37.5%). Furthermore, the identification of potential novel *PCSK9* variants amongst 39% of genetically confirmed FH subjects suggests that this variant is common amongst clinically diagnosed FH in the Malaysian community. Future studies are warranted for the potentially novel variants and the VUS with a strong suggestion of PV, which may need carefully examined further with molecular docking simulation, binding affinity and functional studies. The similar distribution of PV variants of *LDLR* and *APOB* in the present study suggests that *APOB*, along with *LDLR,* are common in the Malaysian population. Whether this is unique in some countries of the Asian region remains to be addressed in future studies.

## Figures and Tables

**Table 1:** Clinical characteristics of the familial hypercholesterolaemia (FH) subjects.

Demographic Characteristics	Potential FH (N = 58)	Possible FH (N = 314)	*p*-Value
Age (years), mean ± SD	58.9 ± 9.9	49.6 ± 13.1	0.01 *
Gender, *n* (%)			
Males	26 (44.8)	145 (46.2)	NS
Females	32 (55.2)	169 (53.8)	
BMI (kg/m^2^), mean ± SD	26.7 ± 5.8	27.6 ± 4.9	NS
Overweight and obesity	43 (74.1)	254 (80.1)	0.042
Waist circumference (cm), mean ± SD	89.2 ± 13.2	90.6 ± 11.2	NS
Hip circumference (cm), mean ± SD	100.7 ± 11.9	101.7 ± 10.3	NS
Abdominal obesity (Male ≥ 0.90; female ≥ 0.85)	37 (63.8)	217 (69.1)	NS
Comorbidities, *n* (%)			
Diabetes, *n* (%)	11 (19.0)	37 (11.8)	NS
FPG (mmol/L), mean ± SD	6.1 ± 2.7	6.0 ± 3.0	NS
RPG (mmol/L), mean ± SD	6.6 ± 2.2	6.7 ± 3.8	NS
Hypertension, *n* (%)	29 (50.0)	141 (44.9)	NS
SBP (mm/Hg), mean ± SD	131.1 ± 18.6	132.0 ± 20.7	NS
DBP (mm/Hg), mean ± SD	76.9 ± 10.9	80.5 ± 12.0	NS
Existing personal CVD (%)	8 (13.8)	16 (5.1)	0.01 *
Family history of PCAD, *n* (%)	20 (34.5)	42 (13.4)	0.001 *
Xanthomata, *n* (%)	6 (10.3)	0 (0.0)	0.001 *
Corneal arcus, *n* (%)	44 (75.9)	7 (2.2)	<0.001 *
Lipid profiles, mean ± SD			
TC (mmol/L)	7.4 ± 1.6	7.3 ± 1.0	0.001 *
TG (mmol/L)	1.8 ± 0.7	1.9 ± 0.7	NS
Baseline LDL-C (mmol/L)	6.1 ± 1.4	5.6 ± 0.7	<0.001 *
HDL-C (mmol/L)	1.4 ± 0.4	1.3 ± 0.3	NS

SD = Standard deviation; BMI = Body mass index, FPG = Fasting plasma glucose; RPG = Random plasma glucose; CVD = Cardiovascular disease; PCAD = Premature coronary artery disease; TC = Total cholesterol; TG = Triglyceride; LDL-C = Low-density lipoprotein cholesterol; HDL-C = High-density lipoprotein cholesterol; NS = not significant. * *p*-value < 0.05

**Table 2:** Detection rate of molecular confirmation amongst clinically diagnosed FH, according to Dutch Lipid Clinic Network criteria categories (n = 372).

Category	Total FH Individuals (*n* = 372)	Potential FH Individuals (*n* = 58)	Possible FH Individuals (*n* = 314)
Presence of pathogenic variants in FH genes (*LDLR, APOB, PCSK9*)	82/372 (22.0%)	12/58 (20.7%)	70/314 (22.3%)
Absence of pathogenic variants in FH genes	290/372 (78.0%)	46/58 (79.3%)	244/314 (77.7%)

*LDLR*: Low-density lipoprotein receptor gene; *APOB*: Apolipoprotein B gene; *PCSK9*: Proprotein convertase subtilisin/kexin type 9 gene; *LDLRAP1*: Low-density lipoprotein receptor adaptor protein 1 gene.

**Table 3:** Clinical and genetically confirmed FH prevalence in the community (n = 5130).

Clinically Diagnosed FH Subjects	Clinical Prevalence	Individuals with ≥1 PV in FH Genes	Genetically Confirmed Prevalence
Potential FH only	58/5130 (1.1%–1:88)	12/5130 (0.2%)	1:427
Possible FH only	314/5130 (6.1%–1:16)	70/5130 (1.4%)	1:73
All FH categories(Potential and Possible FH)	372/5130 (7.3%–1:14)	82/5130 (1.6%)	1:63

PV = Pathogenic variants.

**Table 4:** Genotype frequencies of familial hypercholesterolaemia (FH) pathogenic variants (PV) amongst genetically confirmed FH subjects (n = 82).

Genotypes	Total Number of Individuals with PV (*n* = 82)	Percentage (%)
*LDLR* heterozygote	26	31.7
*APOB* heterozygote	18	22.0
*PCSK9* heterozygote	32	39.0
*LDLR* + *APOB* double heterozygous	3	3.7
*LDLR* + *PCSK9* double heterozygous	1	1.2
*APO*B + *PCSK9* double heterozygous	2	2.4
	82/82	100%

*LDLR*: Low-density lipoprotein receptor gene; *APOB*: Apolipoprotein B gene; *PCSK9*: Proprotein convertase subtilisin/kexin type 9 gene.

**Table 5:** Distribution of variants identified in 372 clinically diagnosed FH subjects according to the genes.

Genes	PV (%)	VUS (%)
*LDLR*	18/40 (45.0)	13/71 (18.3)
*APOB*	15/40(37.5)	42/71 (59.2)
*PCSK9*	5/40 (12.5)	12/71 (16.9)
*LDLRAP1*	2/40 (5.0)	4/71 (5.6)
Total	40 (100)	71 (100)

*LDLR*: Low-density lipoprotein receptor gene; *APOB*: Apolipoprotein B gene; *PCSK9*: Proprotein convertase subtilisin/kexin type 9 gene; *LDLRAP1*: Low-density lipoprotein receptor adaptor protein 1 gene; PV = Pathogenic variant; VUS = Variant of unknown significance.

**Table 6:** List of pathogenic variants identified amongst genetically confirmed FH subjects (n = 82).

	Variant				Database		In Silico Predicted Effects			ACMG	References
No.	HGVS	Exon/Intron	Individuals	dbSNP	LOVD	ClinVar	PolyPhen	SIFT	REVEL		
*LDLR* nonsynonymous variants											
1	c.241C>T (R81C)	3	1	rs730882078	LP, P	Conflicting (P,LP,VUS)	probably damaging	Deleterious	Likely disease causing	PV (PM1, PM2, PP2, PP3, PP4, PP5)	[25,26,27]
2	c.301G>A (E101K)	3	4	rs144172724	LP, P	LP, P	probably damaging	Deleterious	Likely disease causing	PV (PS3, PM1, PM2, PP2,PP3, PP4, PP5)	[28,29]
3	c.580A>G (S194G)	4	1	rs373488885	NR	VUS	benign	Tolerated	Likely benign	LP (PM1, PM2, PP2, PP4)	-
4	c.811G>A (V271I)	5	1	rs749220643	VUS	Conflicting (LP,LB)	benign	Tolerated	Likely benign	LP (PM1, PM2, PP2, PP4, PP5)	[30]
5	c.833G>A (G278E)	6	1	-	NR	NR	probably damaging	Deleterious	Likely disease causing	LP (PM1, PM2, PP2, PP3, PP4)	-
6	c.949G>A (E317K)	7	2	rs746834464	LP, P	Conflicting (P,LP,VUS)	probably damaging	Deleterious	Likely disease causing	LP (PM1, PM2, PP2, PP3, PP4, PP5)	[31,32,33]
7	c.1234A>C (M412L)	9	1	rs1225797407	NR	P	benign	Tolerated	Likely benign	LP (PM1, PM2, PP2, PP4, PP5)	-
8	c.1284C>G (N428K)	9	4	rs368708058	P, VUS	CF (LP,VUS)	probably damaging	Deleterious	Likely disease causing	LP (PM1, PM2, PP2, PP3, PP4, PP5)	[34,35,36]
9	c.1289T>G (V430G)	9	1	-	NR	NR	probably damaging	Deleterious	Likely disease causing	LP (PM1, PM2, PM5, PP2, PP3, PP4)	-
10	c.1571T>G (V524G)	10	1	-	LP	LP	probably damaging	Deleterious	Likely disease causing	LP (PM1, PM2, PP2, PP3, PP4, PP5)	[37,38]
11	c.1774G>T (G592W)	12	6	-	NR	NR	probably damaging	Deleterious	Likely disease causing	LP (PM1, PM2, PM5, PP2, PP3, PP4)	-
12	c.1820A>G (H607R)	12	1	rs879255033	LP	LP	probably damaging	Deleterious	Likely disease causing	LP (PM1, PM2, PP2, PP3, PP4, PP5)	[39]
13	c.2383C>G (P795A)	16	1	-	NR	NR	probably damaging	Deleterious	Likely disease causing	LP (PM2, PM5, PP2, PP3, PP4)	-
14	c.2530G>A (G844S)	17	1	rs1555809614	NR	P	probably damaging	Deleterious	Likely disease causing	LP (PM2, PP2, PP3, PP4, PP5)	-
15	c.1217G>A (R406Q)	9	1	rs552422789	LP	LP	probably damaging	Deleterious	Likely disease causing	LP (PM1, PM2, PP2, PP3, PP4, PP5)	[27,40,41]
16	c.1246C>T (R416W)	9	1	rs570942190	LP, P	LP, P	probably damaging	Deleterious	Likely disease causing	PV (PS3, PM1, PM2, PP2, PP3, PP4, PP5)	[25,42,43]
17	c.1867A>G (I623V)	13	2	rs555292896	LB	Conflicting (P,VUS,B,LB)	benign	Tolerated	Likely disease causing	LP (PM1, PM2, PP2, PP4)	[44]
***LDLR*** **splice site variant**											
18	c.1187-2A>G	8	1	rs879254823	LP, P	LP, P	NA	NA	NA	PV (PVS1, PM1, PM2, PP4, PP5)	[45,46]
**No.**	**HGVS**	**Exon/ Intron**	**Individuals**	**dbSNP**	**LOVD**	**ClinVar**	**PolyPhen**	**SIFT**	**REVEL**	**ACMG**	**References**
***APOB*** **nonsynonymous variants**											
1	c.11303T>C (I3768T)	26	2	rs376825639	VUS, P	VUS	probably damaging	Deleterious	Likely benign	LP (PM1, PM2, PP4, PP5)	-
2	c.11006T>G (L3669R)	26	1	-	NR	NR	probably damaging	Deleterious	Likely benign	LP (PM1, PM2, PM5, PP3, PP4)	-
3	c.9533A>C (K3178T)	26	1	-	NR	NR	possibly damaging	Deleterious	Likely benign	LP (PM1, PM2, PP3, PP4)	-
4	c.9107C>A (S3036Y)	26	2	-	NR	NR	probably damaging	Deleterious	Likely benign	LP (PM1, PM2, PP3, PP4)	-
5	c.8287A>C (K2763Q)	26	1	-	NR	NR	probably damaging	Deleterious	Likely benign	LP (PM1, PM2, PP3, PP4)	-
6	c.7975C>T (P2659S)	26	1	-	NR	NR	possibly damaging	Deleterious	Likely benign	LP (PM1, PM2, PP3, PP4)	-
7	c.7828G>C (A2610P)	26	1	-	NR	NR	probably damaging	Deleterious	Likely benign	LP (PM1, PM2, PP3, PP4)	-
8	c.5756T>C (L1919P)	26	1	-	NR	NR	probably damaging	Deleterious	Likely benign	LP (PM1, PM2, PP3, PP4)	-
9	c.4951G>A (G1651R)	26	2	rs748424949	VUS	NR	possibly damaging	Deleterious	Likely benign	LP (PM1, PM2, PP1, PP3, PP4)	-
10	c.4867G>A (G1623S)	26	1	-	VUS	NR	probably damaging	Deleterious	Likely benign	LP (PM1, PM2, PP3, PP4)	-
11	c.1400C>G (A467G)	11	3	rs376602710	LB	VUS	probably damaging	Deleterious	Likely disease causing	LP (PM2, PP3, PP4, PP5)	-
12	c.10579C>T (R3527W)	26	1	rs144467873	P	Conflicting	probably damaging	Deleterious	Likely disease causing	PV (PS3, PM1, PM2, PP3, PP4)	[25,47,48]
13	c.8462C>T (P2821L)	26	2	rs72653095	B, VUS	Conflicting	probably damaging	Deleterious	Likely benign	LP (PM1, PM2, PP3, PP4)	[49]
14	c.7619G>T (G2540V)	26	1	rs571626569	B	Conflicting	possibly damaging	Deleterious	Likely benign	LP (PM1, PM2, PP3, PP4)	-
***APOB*** **frameshift variant**											
15	c.13028_13029del (Y4343)	29	4	rs760832994	NR	CF (LP, VUS)	NA	NA	NA	PV (PVS1, PS4, PM1, PM2, PP4, PP5)	[50]
**No.**	**HGVS**	**Exon/ Intron**	**Individuals**	**dbSNP**	**LOVD**	**ClinVar**	**PolyPhen**	**SIFT**	**REVEL**	**ACMG**	**References**
***PCSK9*** **nonsynonymous variants**											
1	c.323T>G (L108R) (GOF)	2	1	-	NR	LP, P	probably damaging	Deleterious	Likely benign	PV (PS3, PM1, PM2, PP3, PP4, PP5)	[51]
2	c.1493A>C (E498A)	9	31	-	NR	NR	probably damaging	Deleterious	Likely benign	LP (PM1, PM2, PP3, PP4)	-
3	c.1495C>G (R499G)	9	4	-	NR	NR	possibly damaging	Tolerated	Likely benign	LP (PM1, PM2, PM5, PP4)	-
4	c.212C>T (P71L) (GOF)	2	2	rs569379713	LP	VUS	benign	Deleterious	Likely benign	LP (PM1, PM2, PP4, PP5)	[52,53]
5	c.286C>T (R96C) (GOF)	2	1	rs185392267	VUS	Conflicting	probably damaging	Deleterious	Likely benign	PV (PS3, PM1, PM2, PP3, PP4, PP5)	[53,54]
*LDLRAP1* nonsynonymous variants *											
1	c.281C>A (P94Q)	3	2	-.	NR	NR	probably damaging	Deleterious	Likely benign	LP (PM2, PM3, PP3, PP4)	-
***LDLRAP*** 1 frameshift variants *											
**No.**	**HGVS**	**Exon/ Intron**	**Individuals**	**dbSNP**	**LOVD**	**ClinVar**	**PolyPhen**	**SIFT**	**REVEL**	**ACMG**	**References**
2	c.604delT	6	1	-	NR	P	NA	NA	NA	PV (PVS1, PM1, PM2, PM3, PP5)	[55,56]

*LDLR:* Low-density lipoprotein receptor gene; *APOB:* Apolipoprotein B gene; *PCSK9:* Proprotein convertase subtilisin/kexin type 9 gene; *LDLRAP1:* Low-density lipoprotein receptor adaptor protein 1 gene; P = Pathogenic; LP = Likely pathogenic; VUS = Variant of unknown significance; B = Benign; LB = Likely benign; NR = Not reported; NA = Not available; PVS = Very strong evidence; PS = Strong evidence; PM = Moderate evidence; PP = Supporting evidence. * Autosomal recessive variants which may only exert the pathogenicity in homozygous form.

**Table 7:** Prevalence of genetically confirmed familial hypercholesterolaemia amongst the general population in different countries.

Country	Data Source	Prevalence	Reference
United Kingdom	Population-based	1:252	[67]
United States	Genomic sequencing and HER data	1:222	[40]
Japan	Children screening program	1:617	[68]
Denmark	Population-based	1:575	[69]
Finland	National FINRISK Study and the Health 2000 Cohort Studies	1:813	[70]
Switzerland	Population-based	1:549	[71]

## Data Availability

Not applicable.

## Supplementary Materials

The following supporting information can be downloaded at: https://www.mdpi.com/article/10.3390/ijms232314971/s1.

## Appendix A

Malaysian HEalth and Well-Being AssessmenT (MyHEBAT)—Familial Hypercholesterolaemia (FH) research investigators.

**Universiti Teknologi Mara (UiTM):** Hapizah Nawawi (I-PPerForM, Faculty of Medicine and Al-Sultan Abdullah Hospital), Anis Safura Ramli (I-PPerForM and Faculty of Medicine), Suraya Abdul Razak (I-PPerForM and Faculty of Medicine), Noor Alicezah Mohd Kasim (I-PPerForM, Faculty of Medicine and Al-Sultan Abdullah Hospital), Thuhairah Hasrah Abdul Rahman (I-PPerForM, Faculty of Medicine and Al-Sultan Abdullah Hospital), Noor Shafina Mohd Nor (I-PPerForM, Faculty of Medicine and Al-Sultan Abdullah Hospital), Alyaa Al-Khateeb (I-PPerForM and Faculty of Medicine), Suhaila Abd Muid (I-PPerForM and Faculty of Medicine), Yung-An Chua (I-PPerForM), Aimi Zafira Razman (I-PPerForM), Sukma Azureen Nazli (I-PPerForM), Zaliha Ismail (Faculty of Medicine), Salmi Razali (I-PPerForM, Faculty of Medicine and Al-Sultan Abdullah Hospital), Muthukkaruppan Annamalai (Faculty of Computer Science and Mathematics), Marshima Mohd Rosli (I-PPerForM and Faculty of Computer Science and Mathematics), Siti Hamimah Sheikh Abdul Kadir (I-PPerForM and Faculty of Medicine).
