# Supplementary material for: Genetic Spectrum of Familial Hypercholesterolaemia in the Malaysian Community: Identification of Pathogenic Gene Variants Using Targeted Next-Generation Sequencing
Source: Int J Mol Sci. 2022 Nov 29;23(23):14971. doi: 10.3390/ijms232314971 (PMC9736953; doi:10.3390/ijms232314971)
Supplement: Supplementary file 1 [file ijms-23-14971-s001.zip › ijms-1968050-supplementary.pdf]

Supplementary Materials

Several variants in the exon 9 of *PCSK9* are projected on the crystallographic structure **(Supplementary Figure S1).**

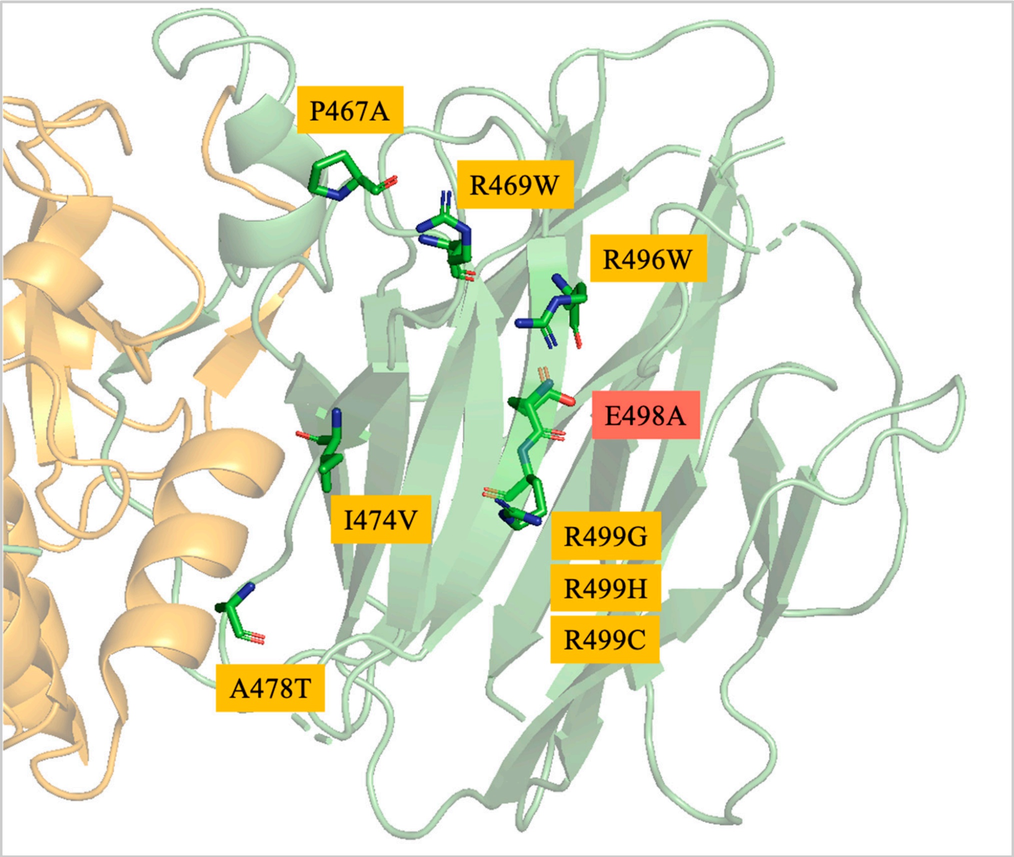

Supplementary Figure S1 A list of *PCSK9* variants located on exon 9 (reviewed by https://doi.org/10.3390/pr9020283) (yellow boxes), and E498A (yellow box) revealed in this study**.** The wildtype residues (shown as sticks) representing the variants are projected on the crystallographic structures of PCSK9 (PDB ID 3P5C).

The other two missense pathogenic variants (PV) of *PCSK9* located at exon 9, namely *PCSK9*: c.1493A>C (E498A) and c.1495C>G (R499G), have not been reported in any literature or database. The former PV was found in 31 individuals, which majority of the subjects (27/31) do not have any other PV in other FHCG reported in this study. Furthermore, *in silico* finding in this present study highly suggests a damaging effect of the variant that could affect the protein function. The c.1493A>C (E498A) variant is located in the C-terminal domain of *PCSK9*, which responsible in increasing the affinity between PCSK9 and LDL receptor at the low pH of the endosomes. To support the pathogenicity of this variant, a structural analysis of E498A was conducted and projected on the crystallographic structure of the *PCSK9* (PDB ID 3P5C) . Based on this experimental structure, the wildtype residue, E498 is shown to form hydrogen bond interactions with two serine residues, S488 and S564. However, for the E498A variant, computational mutagenesis predicts the replacement of glutamic acid (E), which is a negatively charged amino acid to an alanine (A), a small hydrophobic amino acid that cause a loss of interaction with one of the serine molecules. Hence, the disruption of the intramolecular interactions may affect the overall structural conformation of the *PCSK9* C-terminal domain (**Supplementary Figure S2)**.

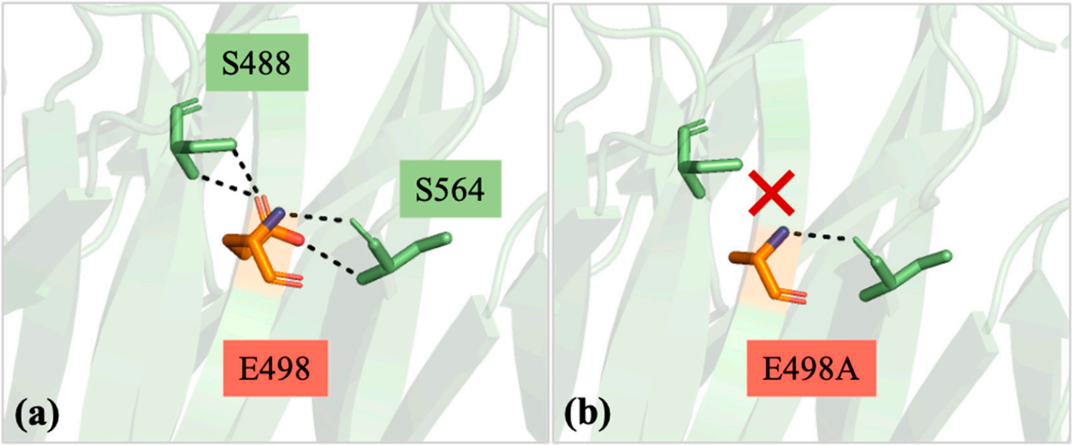

Supplementary Figure S2 Structural analysis of the novel E498A variant projected on the crystallographic structures of PCSK9 (PDB ID 3P5C). (a) The wildtype residue, E498 is shown to form hydrogen bond interactions with two serine residues, S488 and S564. (b) Computational mutagenesis predicts the replacement of glutamic acid, which is a negatively charged amino acid to an alanine, a small hydrophobic amino acid that will cause a loss of interaction with S488.

The second variant, *PCSK9*:c.1495C>G (R499G) was found in four subjects and this variant has pathogenicity evidence of PM1, PM2, PM5 and PP4, which classified this variant as likely pathogenic according to ACMG guidelines. Initially, *in silico* finding of this variant from this present study revealed that it is less likely to be damaging. However, based the experimental structural analysis of the wild-type, R499 is shown to form salt-bridge (ionic) interactions with E501 and hydrogen bonds with R510. Computational mutagenesis of R499G, revealed the ionic interaction between the variant and residue E501 is abolished due to the change of amino acid. The same scenario occurs with the previously reported PV of *PCSK9*, R499H [99]. The abolishment of the ionic interaction highly likely will disrupt the structure of the C-terminal domain of *PCSK9*, hence may affect protein function (**Supplementary Figure S3**).

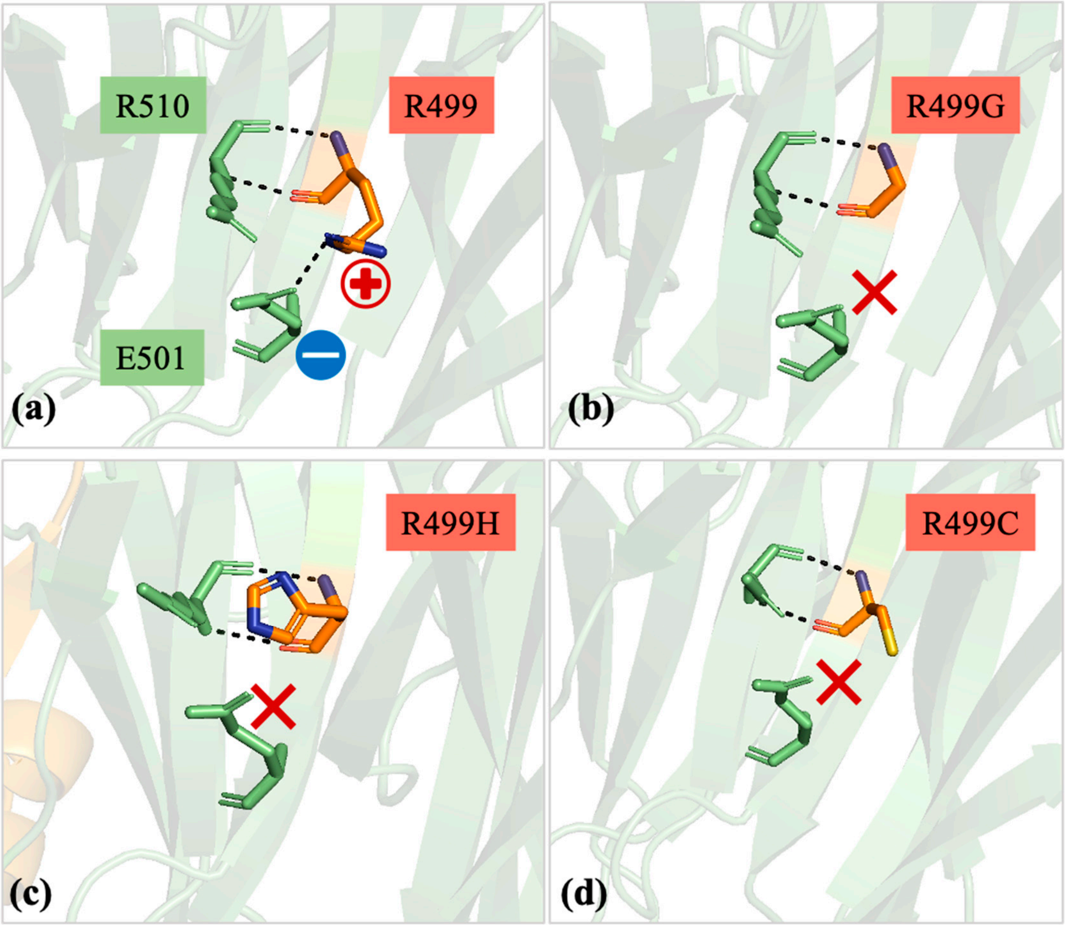

Supplementary Figure S3 Structural analysis of variants at R499 projected on the crystallographic structures of PCSK9 (PDB ID 3P5C). The wildtype amino acid, R99 is shown to form salt-bridge (ionic) interactions with E501, and hydrogen bonds with R510. Computational mutagenesis of R499G, R499H and R499C shows the ionic interaction between the variant and residue E501 is abolished due to the change of amino acid.

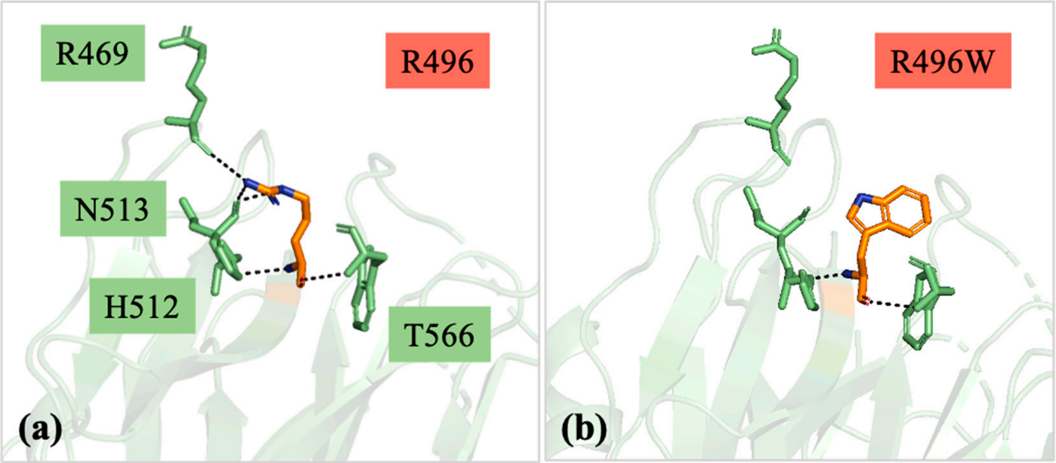

Supplementary Figure S4 Structural analysis of the R496W variant projected on the crystallographic structures of PCSK9 (PDB ID 3P5C). (a) The wildtype amino acid, R496 forms polar interactions with four residues: H512, R469, N513, and T566. (b) Computational mutagenesis shows that the R496W variant caused loss of interactions with two residues. In addition, the replacement of tryptophan, which is hydrophobic and has a bulky aromatic side-chain may cause a collision with nearby residues that highly likely will disrupt the structure of the C-terminal domain of PCSK9.

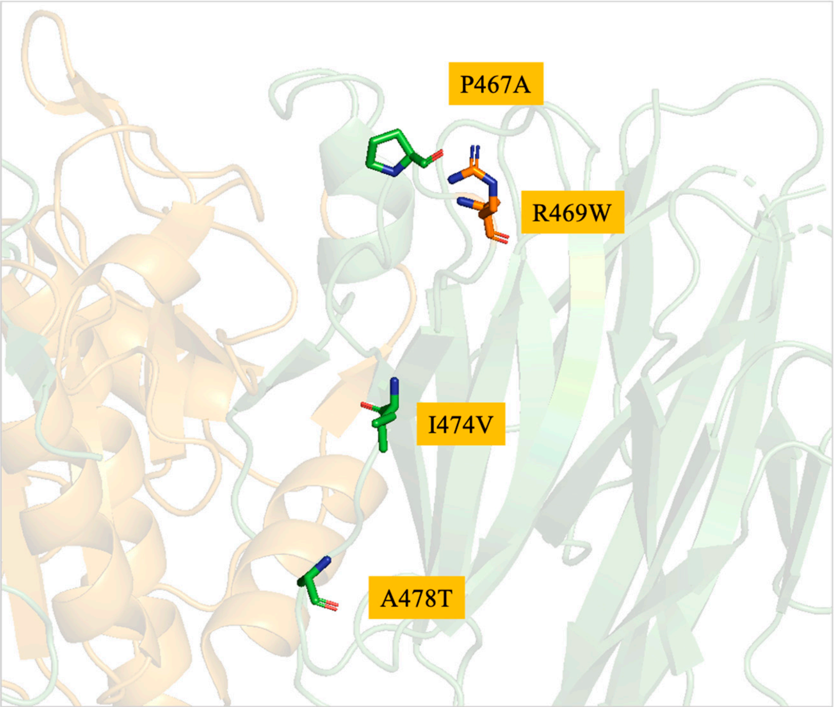

Supplementary Figure S5 Structural analysis for P467A, R469W, I474 and A478T variants projected on the crystallographic structures of PCSK9 (PDB ID 3P5C). The wildtype amino acids of these variants have no polar interaction with the surrounding amino acids. Thus, there will not be much impact on the structure caused by the variants, especially for the I474V and A478T variants that are replacing similar types of amino acids.
